# JNK mediates serine phosphorylation of STAT3 in response to fatty acids released by lipolysis

**DOI:** 10.21203/rs.3.rs-6150649/v1

**Published:** 2025-03-05

**Authors:** Ayla Melisa Aksu, Amena Akter, Preetveer Dhillon, Zane J. Zerbel, Pania E. Bridge-Comer, Oluwafemi Gbayisomore, Shannon M Reilly

**Affiliations:** Weill Cornell Medicine; Cornell; Weill Cornell Medicine; Weill Cornell Medicine

**Keywords:** Adipocyte, Adipose tissue, Cell signaling, Lipolysis and fatty acid metabolism, Lipid droplets, Obesity

## Abstract

Adipocytes play an essential role in energy balance and metabolic health. Excess nutrients are stored within the white adipose tissue (WAT) as triglycerides. Energetic demand is communicated to the adipocyte by the sympathetic nervous system. Catecholamines released by nerve terminals in the adipose tissue promote lipolysis, a process in which triglycerides are broken down into fatty acids and glycerol. Lipolytic activation of white adipocytes is associated with an increase in the rate of oxygen consumption. This lipolysis induced respiration requires phosphorylation of signal transducer and activator of transcription 3 (STAT3) at Ser^727^. This study identifies c-Jun N-terminal kinase 1 (JNK1) as the kinase responsible for this critical phosphorylation event, and thus a key regulator of lipolysis-driven oxidative metabolism. We demonstrate that JNK1 is activated in response to intracellular fatty acids released during lipolysis and phosphorylates lipid droplet-associated STAT3, leading to inhibition of glycerol-3-phosphate acyltransferase 3 (GPAT3) and suppression of fatty acid re-esterification. This mechanism promotes uncoupled mitochondrial respiration, increasing energy expenditure. Inhibition of JNK1 attenuated oxidative metabolism without affecting the rate of lipolysis. The MAP kinase cascade upstream of JNK1 in lipolytic adipocytes remains unclear. Neither apoptosis signal-regulating kinase 1 (ASK1) nor mitogen-activated protein kinase kinases 4/7 (MKK4/7) appear to be required. Our findings suggest that JNK1 functions as a metabolic sensor in adipocytes, activating oxidative metabolism through STAT3 phosphorylation in response to fatty acids, with implications for energy balance and obesity-related metabolic regulation.

## Introduction

Obesity is a medical condition marked by the excessive accumulation of body fat, which is linked to serious health issues including heart disease, type 2 diabetes, inflammation, and specific cancers. In individuals with obesity, particularly those with an excess of visceral fat, immune cells in adipose tissue are activated. These cells secrete pro-inflammatory cytokines, contributing to a state of low-grade, chronic inflammation. Chronic inflammation has been implicated in the pathology of obesity-related diseases. Conversely, acute inflammation increases energy expenditure and may be an early adaptive response to obesity^1^. Acute inflammation triggers a metabolic shift characterized by increased energy expenditure resulting from immune system activation, fever, and altered nutrient utilization^2^.

Excess nutrients are stored as triglycerides within white adipose tissue (WAT). Fatty acids undergo esterification onto a glycerol backbone via the glycerol lipid synthesis pathway. The first and rate-limiting enzyme in this pathway in WAT is glycerol-3-phosphate acyltransferase 3 (GPAT3). During stress, such as fasting or cold exposure, these lipid stores can be mobilized in response to the sympathetic nervous system. Local secretion of catecholamines within adipose tissue activates lipolysis and the release of triglyceride stores as fatty acids and glycerol. However, not all fatty acids released by lipolysis enter the circulation; at least one-third are retained within WAT. Typically, white adipocytes have a strong preference for esterification and storage of fatty acids. However, during lipolysis enzymatic activity of the esterification pathway is suppressed^3–5^, resulting in increased oxidative metabolism. One mechanism by which adipocytes suppress esterification during lipolysis is via serine phosphorylation of STAT3. The elevation in intracellular fatty acids during lipolysis stimulates Ser^727^ phosphorylation of lipid-droplet associated STAT3, increasing its interaction with and suppression of GPAT3 activity. Thus, Ser^727^ phosphorylation of STAT3 is essential for the induction of lipolysis driven oxidative metabolism^3^.

The protein kinases that phosphorylate STAT3 at Ser^727^ have been the subject of much investigation^6–14^.. Proline residues surrounding the Ser^727^ site match the MAPK consensus sequence [P-X-S/T-P]^15–17^. In this regard, p38, ERKs and JNKs have been implicated as STAT3 serine kinases^6, 7, 13, 18–20^. Here we investigate the possibility that JNK is the kinase responsible for STAT3 Ser^727^ phosphorylation in response to lipolysis. Importantly, JNK has been observed to directly phosphorylate STAT3 in an *in vitro* kinase assay^16^. Catecholamine-stimulated JNK activity has also been shown to depend on fatty acid reuptake, consistent with activation of JNK downstream of fatty acids^21–23 24^. Here we investigate whether JNK may be the kinase that phosphorylates STAT3 at Ser^727^ in response to fatty acids released during lipolysis.

## Materials and Methods

### Reagents

The following reagents were used in this study: Amphotericin B (Sigma, A2411), Atglistatin (ATGLi, Sigma SML1075), Bovine Serum Albumin (BSA, Sigma, A028), BSJ-04–122 (MKK4/7, MedChemExpress, HY-152185), Carbonyl cyanide 4 (trifluoromethoxy)phenylhydrazone (FCCP, Sigma C2920), CL-316,243 (CL, Sigma C5979), Collagenase (Sigma – C6885), DB07268 (JNK1 inhibitor, MedChemExpress, HY-15737), Dexamethasone (Sigma D4902), DMEM/F-12 50/50 (Corning 15–090), Extracellular matrix gel from Engelbreth-Holm-Swarm murine sarcoma (Sigma E1270), Fetal bovine serum (FBS, Corning 35 − 010), Fibronectin (Sigma F1141), GS-444217 (ASK1 inhibitor, MedChemExpress, HY-100844), Insulin (Sigma I6634), 3-Isobutyl-1-methylxanthine (IBMX, Sigma I5879), MitoTracker green (Fisher M7514), Oligomycin A (Sigma 75351), Pen Strep Glutamine (PSG, Gibco 10378–016), PF-04620110 (DGATi, Sigma PZ0207), Rosiglitazone (Sigma 557366), Rotenone (Sigma R8875), SP600125 (JNK inhibitor, MedChemExpress, HY-12041), TCSJNK5a (JNK IX inhibitor, Selleckchem, S7508), Tetramethylrhodamine, methyl ester (TMRM, Fisher I34361), Triacsin C (Sigma T4540).

### Animals

Animals homozygous for the *Stat3* floxed allele (Stock No: 016923) were bred to *Adipoq*-promoter driven *Cre* mice (Stock No: 028020) to generate mice homozygous for the *Stat3* floxed allele both with and without the *Adipoq-Cre*. Animals with *Adipoq-Cre* expression lose *Stat3* in mature adipocytes and are referred to in the manuscript as SAKO animals, while floxed littermate controls without *Adipoq-Cre* are referred to as SAWT.

All strains of mice were on the C57BL/6J background (Stock No: 000664). Animals for experiments were bred in-house. Animals in each cohort were produced from multiple breeding pairs to minimize the birth date range. Extra attention was paid to housing arrangements to ensure that each cage accommodated multiple treatment groups, to minimize potential confounding by the cage effect. During animal studies, ear tag numbers were used to identify animals. Within an experiment, the genotype and/or treatment groups were both littermates and cage mates. Researchers performing tests and collecting data were blinded during experiments. Sample sizes were determined using a power analysis with the expected effect size but were sometimes limited by availability. Sex as a biological variable was considered; female and male cohorts were analysed separately.

Mice were housed in a specific pathogen-free facility with a 12-hour light-dark cycle and were given free access to food and water. Mice were fed a normal diet (5053, Labdiet Picolab). All animal use was approved by the Institutional Animal Care and Use Committee (IACUC) at Weill Cornell Medicine.

### Cell Culture

#### Primary adipocytes:

Primary preadipocytes were isolated from inguinal fat pads as follows: Following fine mincing, the tissue was digested with 1 mg/ml collagenase and 2% BSA in a 37°C water bath with shaking for 20–35 min. The digestion was stopped by adding 15% FBS media 1:1 to the serum-free collagenase media, then the slurry was passed through a 100 μm filter and spun at 500 g for 5 min. The pellet was washed and resuspended in culture media (DMEM/F-12 with 15% FBS and PSG) and plated with 2.5 mg/L amphotericin B and placed in a 10% CO_2_ incubator. Nonadherent cells were washed away three days later. When the cells reached ~ 80% confluence, they were passed from their original 10 cm culture plate to a 15 cm plate and incubated for an additional 2–5 days. Cells from the second passage were plated to confluence for experiments on extracellular matrix and fibronectin coated plates. Differentiation was initiated with 500 μM 3-Isobutyl-1-methylxanthine, 5 μM dexamethasone, 1 μg/ml insulin and 1 μM rosiglitazone for 3 days, followed by insulin alone for at least 3 days. Cells were used for experiment 7–10 days after the initiation of differentiation. Twenty-four hours prior to the start of an assay, insulin was removed from the culture media. Only cultures in which > 90% of cells displayed adipocyte morphology were used.

#### Fractionation:

adipocytes were fractionated using differential centrifugation in fractionation buffer: HEPES (pH 7.4) 20 mM, KCI 10 mM, MgCl2 2 mM, EDTA 1 mM, EGTA 1 mM, DTT 200 μM, NaF 10 mM, NaVO4 1 mM, β-glycerophosphate 2.5 mM, phosphatase inhibitor tablet (Roche). All steps were performed at 4°C. Cells and tissues were lysed with a glass dounce homogenizer, then spun at 1,000g for 10 min to separate lipid droplets (floating fat cake) and nuclear pellets. Mitochondria were then pelleted from the supernatant at 10,000g, and then membrane was pelleted at 21,000g after incubation with 8 mM CaCl2. The remaining proteins in the solution were cytosol. Each fraction (except cytosol) was resuspended in a fractionation buffer and spun a second time to wash, before addition of a lysis buffer. Each supernatant was spun a second time at the same speed, before being transferred to a new tube to pellet the next fraction. Protein content in each fraction was normalized, and 20 μg of protein was loaded per well for western blot analysis.

#### GPAT activity assay:

GPAT-specific activity was assayed for 15 min at room temperature in a 200 μl reaction mixture containing 100 μg total lysate protein in GPAT assay buffer (75 mm Tris-HCl, pH 7.5, 4 mm MgCl2, 1 mg ml–1 BSA (FA-free), 1 mm dithiothreitol, 8 mm NaF, 77 μM (2 μCi per reaction) glycerol 3-phosphate and 50 μM lauryl-CoA). PPDIV cell lysates were prepared by 10 passages through a 28-G needle in GPAT assay lysis buffer (250 mM sucrose, 10 mM Tris pH 7.5, 1 mM EDTA and 8 mm NaF). iWAT tissue lysates were prepared by mechanical homogenization (Roto Star) with 1 μl GPAT assay lysis buffer per mg tissue. Crude lysates were spun at 1,000g for 10 min, followed by 17,000g for 15 min. Supernatant was transferred to a new tube, and protein concentration was measured before dilution to 1 mg ml–1 and addition of 2Å~ GPAT assay buffer. NEM treatments were performed for 15 min prior to addition of 2Å~ GPAT assay buffer. Reactions were terminated by addition of 2:1 chloroform: methanol to extract lipids and precipitate protein. Lipid extraction was repeated an additional two times, and lipids were dried and separated by TLC with 6:35:8 chloroform: methanol: water, to isolate phosphatidic acid. The spot corresponding to phosphatidic acid was identified based on a standard 16:0PA standard (Avanti Polar Lipids, 830855) and visualized with I2 to be scraped off the plate and radioactivity quantified in a liquid scintillation counter (Perkin Elmer, MicroBeta TriLux 1450).

#### Seahorse respiration assays:

Extracellular oxygen consumption rates were measured with a Seahorse XFe96 analyser. Primary preadipocytes were differentiated in 96 well Seahorse XFe96 culture plates. After differentiation, cells were switched to Seahorse XF base DMEM (Agilent 102353) supplemented with 2 mM glutamine, 1 mM pyruvate, and 8 mM glucose. Pre-treatments were added to the base medium. During the assays, drug treatments were injected sequentially using the ports. Unless otherwise indicated, Port A contained 100 nM CL-316,2 or vehicle control, Port B: 2 mM oligomycin, Port C: 1 mM FCCP, and Port D: 1 mM rotenone and 1 mM antimycin A.

### Mitochondrial membrane potential

Tetramethylrhodamine, Methyl Ester, Perchlorate (TMRM) staining was performed and imaged with an imageXpress MICRO Confocal Automated High-Content Analysis System to visualize live mitochondria membrane potential. Cells were stained with 200 nM TMRM thirty minutes, then washed three times with PBS. Imaging was performed in a live cell imaging solution (Invitrogen A59688DJ). After baseline images were obtained, cells were treated with either vehicle control or CL-316,243. Finally, cells were treated with 1 mM FCCP as a negative control form membrane potential.

### Lipolysis assay

FA concentration was measured using 10 μl conditioned media with the NEFA kit (WAKO), using 75 μl Reagent A and 150 μl Reagent B. Absorbance was measured at 550 nm (reference 660 nm) using the manufacturer’s protocol. Free glycerol concentration was measured by reacting 25 μl of sample with 175 μl Free Glycerol Reagent (Sigma), and absorbance was measured at 540 nm using the manufacturer’s protocol.

#### Western blot analysis:

We homogenized tissues and cells in lysis buffer (50 mM Tris pH 7.5, 150 mM NaCl, 2 mM EDTA, 10% glycerol, 1% Triton X-100, 1 mM dithiothreitol, 1 mM Na3VO4, 5 mM NaF, 1 mM phenylmethanesulfonyl fluoride, 25 mM glycerol 2-phosphate and freshly added protease inhibitor tablet) and then incubated them for 1 hour at 4°C. We centrifuged crude lysates at 17,000g for 15 min twice and determined the protein concentration using Bio-Rad Protein Assay Dye Reagent. Mature adipocytes were isolated using the same protocol as that used to isolate preadipocytes for PPDIVs, except that 3 spins were done at 20g to wash the floating mature adipocytes, followed by protein precipitation with 5% TCA, acetone wash and resuspension in 8 M urea, 100 mM Tris pH 8.0. Samples were diluted in the SDS sample buffer. Bound proteins were resolved by SDS–PAGE and transferred to nitrocellulose membranes (Bio-Rad). Individual proteins were detected with specific antibodies and visualized on film using horseradish-peroxidase-conjugated secondary antibodies (Bio-Rad) and Western Lightning Enhanced Chemiluminescence (Perkin Elmer Life Sciences). Primary antibodies against the following were used at a 1:1,000 dilution unless otherwise specified and were purchased from Cell Signaling: JNK (9252), pS563 HSL (4139), HSL (4107), p727 STAT3 1:500 (9134), STAT3 1:4,000 (9139), pS73 (9164), c-JUN (9165), pJNK (9251), pT183/Y185. Goat anti-mouse (31430) and goat anti-rabbit (31460) secondary antibodies were purchased from Thermo Fisher, and were used at a concentration of 1:10,000. Uncropped western blot scans with the size markers indicated are included in the Source Data files. All blots are from separate membranes unless otherwise noted, but were run, transferred and blotted in parallel using the same power source and antibody dilutions.

### Statistical analysis

Two-way or one-way analysis of variance (ANOVA) was performed to evaluate statistical significance, followed by the Holm-Sidak post-hoc analysis to determine specific between-group and time-dependent differences. In each case, significance was set at α = 0.05. Statistical analyses were performed in GraphPad Prism version 10.

## Results

**Figure 1:**
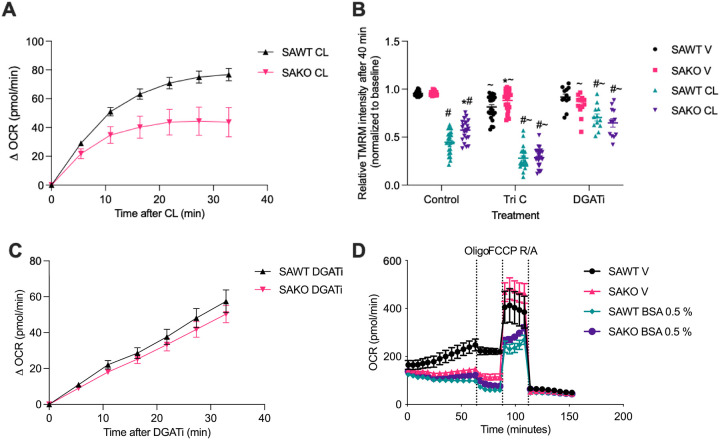
Increased respiration in lipolytic adipocytes is dependent on STAT3 suppression of esterification. **A** Change in oxygen consumption rate (OCR) from baseline after stimulation with vehicle or CL-316,243 at 100 nM in differentiated primary adipocytes isolated from adipocyte-specific knockout (SAKO) mice and floxed littermate control (SAWT) mice. **B** Relative TMRM staining intensity normalized to baseline, 5 ìM triacsin C (ACSL inhibitor) and 10 ìM PF-04620110 (DGAT inhibitor) primary adipocytes after 40-minute stimulation with 100 nM CL or vehicle. **C** Change in oxygen consumption rate (OCR) from baseline after stimulation with vehicle or 10 ìM PF-04620110 (DGAT inhibitor) in differentiated primary adipocytes isolated from adipocyte-specific knockout (SAKO) mice and floxed littermate control (SAWT) mice. **D** OCR in SAKO and SAWT adipocytes in the presence or absence of 0.5% BSA in the media. Treatments: 2 mM oligomycin (oligo), 1 mM FCCP, and 1 mM rotenone/antimycin A (R/A). *p-val < 0.05 SAWT vs. SAKO, ^#^p-val < 0.05 V vs. CL, ^~^p-val < 0.05 vs. Control. n = 3.

Cellular assays were performed using adipocyte progenitor cells differentiated in vitro, which have high lipolytic activity and exhibit robust lipolysis induced oxidative metabolism. Consistent with previous reports, stimulation of lipolysis with the β−3 adrenergic receptor agonist, CL-316,243, resulted in a rapid increase in oxygen consumption rate that was blunted in STAT3 knockout adipocytes (Fig. 1A). STAT3 promotes oxidative metabolism by inhibiting fatty acid esterification via GPAT3. Although triglyceride cycling creates ATP demand it is not the driver of oxidative metabolism during adipocyte lipolysis^3^. Rather, elevated intracellular fatty acid levels drive respiration by promoting AAC mediated uncoupling. The first step in fatty acid re-esterification is fatty activation by long chain acyl-CoA synthetase (ACSL), while diacylglycerol acyl transferase (DGAT) catalyzes the final step of triglyceride synthesis. Inhibition of fatty acid re-esterification via either ACSL or DGAT increases uncoupling resulting in a reduction in mitochondrial membrane potential (Fig. 1B). Consistent with inhibition of re-esterification by STAT3 promoting uncoupled respiration, STAT3 knockout adipocytes displayed attenuated mitochondrial depolarization that was corrected by inhibition of re-esterification (Fig. 1B). Further supporting that the defect in STAT3 KO adipocytes is related to the suppression of esterification, no difference in the induction of oxidative metabolism due to DGAT inhibition was observed between STAT3 KO and WT control adipocytes (Fig. 1C). Finally, sequestration of free fatty acids into the media by albumin suppressed respiration in WT adipocytes to the level of STAT3 KO adipocytes, supporting the role of fatty acids. Together, these data are consistent with suppression of re-esterification by STAT3 as an essential component of lipolysis driven respiration.

**Figure 2:**
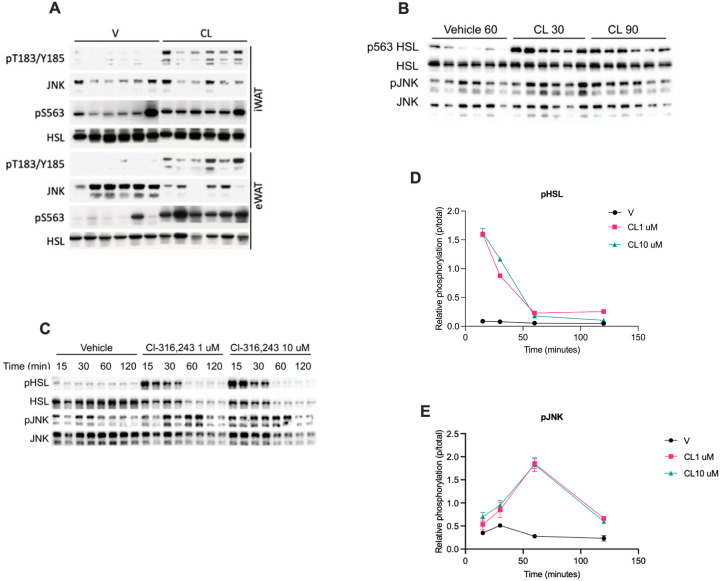
JNK is activated by lipolysis in adipocytes. **A** Western blot analysis of inguinal white adipose tissue (iWAT) and epididymal white adipose tissue (eWAT) from mice stimulated with a 1 mg/kg dose of CL-316,243 (CL) or vehicle via intraperitoneal (IP) injection for 20 minutes. **B** Western blot analysis of eWAT from mice stimulated with a 1 mg/kg dose of CL-316,243 (CL) via IP injection for 30 and 90 minutes or vehicle injection for 60 minutes. **C** Western blot analysis was performed on treated PPDIVs following stimulation with 1 μM and 10 μM of CL-316,243 (CL) or vehicle. **D, E** Quantification of western blots in 2C.

In response to elevated intracellular fatty acids during lipolysis, we have observed increased Ser^727^ phosphorylation of STAT3. This phosphorylation site matches the MAPK consensus sequence, which in combination with sensitivity to fatty acids lead us to investigate the role of c-Jun activated kinase (JNK). First, we investigated whether lipolysis activates JNK. In vivo adipose tissues exhibited increased JNK phosphorylation in response to lipolytic activation by CL-316,243 (Fig. 2A and B). In cultured adipocytes, JNK activation was observed upon lipolytic activation, peaking at one hour in contrast with HSL phosphorylation which occurs rapidly then subsides (Fig. 2C). These data suggesting JNK activation by lipolysis are consistent with previous reports in 3T3-L1 adipocytes as well as other mouse strains^24^.

**Figure 3:**
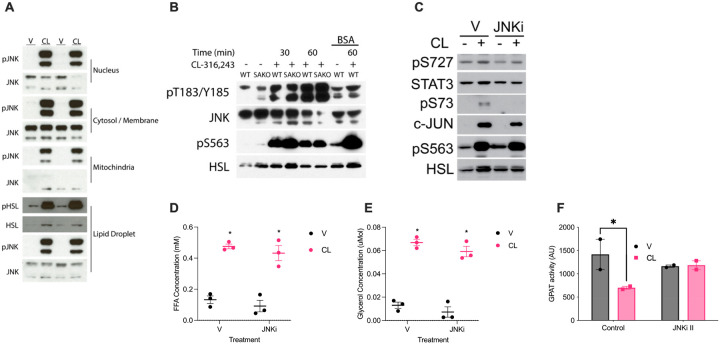
JNK activity is required for STAT3 Ser^727^ phosphorylation and GPAT inhibition. **A** Western blot analysis was performed on fractionated PPDIVs following stimulation with 1 μM CL-316,243 (CL) or vehicle for 15 minutes. **B** Treatment of PPDIVs from SAWT or SAKO mice with and without 2% BSA and CL-316,243 at 30 and 60 minutes or vehicle. **C** Western blot analysis was performed on fractionated lipid drop (LD) PPDIVs following stimulation with 1 μM CL-316,243 (CL) or vehicle for 15 minutes. **D** Rate of free fatty acid (FFA) release from 3T3-L1 and primary adipocytes pretreated with 20 μM JNKi or vehicle, then stimulated with 100 nM CL-316,243 or vehicle. *p-val < 0.05 V vs. CL **E** Rate of glycerol release. *p-val <0.05 V vs. CL **F** GPAT activity assay on the in vitro differentiated adipocyte with 1 μM CL-316,243 (CL) or vehicle and 20 μM JNKi II or vehicle. *p-val < 0.05 V vs. CL

Although Ser^727^ phosphorylation of lipid droplet associated STAT3 increases with lipolysis, no change in STAT3 localization to the lipid droplet is observed, suggesting that the kinase that phosphorylates STAT3 does so in the vicinity of the lipid droplet. Thus, we assayed the subcellular localization of JNK. We found JNK in all fractions, with the highest levels in the cytosolic fraction and the lowest in the mitochondrial fraction (Fig. 3A). Furthermore, all fractions, including the lipid droplet associated fraction, exhibited a robust increase in JNK phosphorylation in response to CL-316,243 (Fig. 3A). These data indicate that JNK is activated at the lipid droplet where STAT3 is phosphorylated. Furthermore, JNK activation was observed to occur normally in STAT3 knockout adipocytes but was suppressed by fatty acid sequestration in the media by BSA (Fig. 3C). These results are consistent with JNK activation upstream of STAT3. Inhibition of JNK attenuated Ser^727^ phosphorylation of STAT3 in the lipid droplet fraction. The efficacy of JNK inhibition was confirmed by loss of c-JUN phosphorylation at serine 73. Importantly, inhibition of JNK did not impact the rate of either fatty acid or glycerol release from adipocytes (Fig. 3D and E).

**Figure 4:**
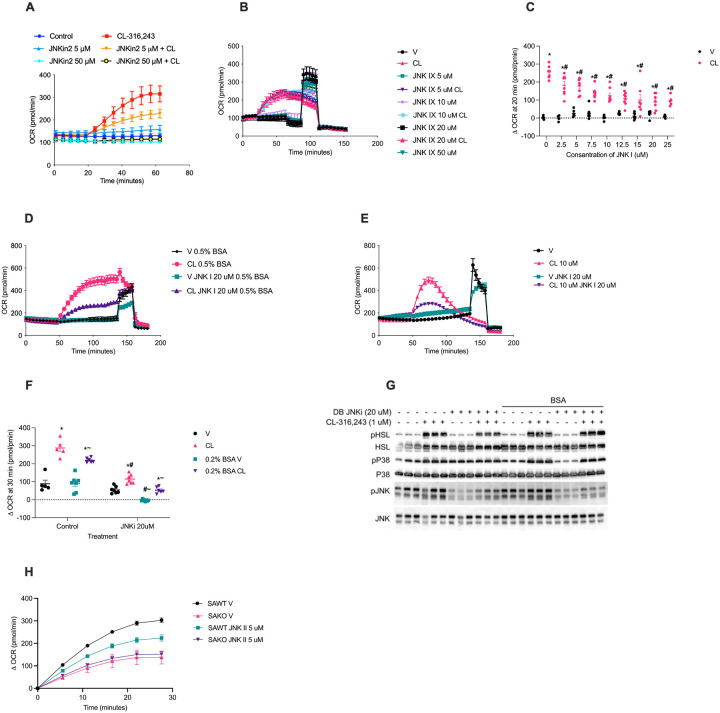
JNK1 activity is required for lipolysis-driven oxidative metabolism. **A** Oxygen consumption rate (OCR) in primary adipocytes pretreated with 5 μM and 50 μM SP600125 at the 15-minute time point before baseline measurements. Port A: 0.5 μM CL-316,243 (CL) or vehicle control. **B** OCR in primary adipocytes pretreated with 5 μM, 10 μM, 20 μM, and 50 μM JNK IX at the 15-minute time point before baseline measurements. Port A: 100 nM CL-316,243 (CL) or vehicle control. **C** Change in OCR in primary adipocytes following a 15-minute pretreatment with DB07268 at the indicated doses, measured from baseline to 20 minutes after stimulation with CL. *p-val < 0.05 V vs. CL, ^#^ p-val < 0.05 vs. Control. n = 6–8. **D** OCR in 0.5% BSA media, with pre-treatment using 20 μM JNK I inhibitor or vehicle, followed by stimulation with 10 μM CL or vehicle in Port A. **E** OCR in primary adipocytes pretreated with 20 μM JNK I inhibitor or vehicle, then stimulated with 10 μM CL or vehicle in Port A. **F** Change in OCR from baseline to 30 minutes after stimulation with 100 nM CL or vehicle control in primary adipocytes, following varying durations in 2% BSA or vehicle media. *p-val < 0.05 V vs. CL, ^#^p-val < 0.05 V vs. JNKi, ^~^p-val < 0.05 V vs. BSA. **G** Wester blot analysis with and without BSA, after stimulation of 1 μM CL-316,243 (CL) or vehicle and, 20 μM JNKi or vehicle. **H** Change in OCR in primary adipocytes from SAKO and littermate control SAWT mice following stimulation of a 5 μM JNKII inhibitor or vehicle.

We have previously demonstrated that Ser^727^ phosphorylation of STAT3 is essential for its interaction with GPAT3, which suppresses esterification and drives respiration^3^. Consistently, the suppression of GPAT activity observed upon lipolytic activation in adipocytes, was not observed in the presence of JNK inhibition (Fig. 3F). Thus, we hypothesized that inhibition of JNK blocks lipolysis driven oxidative metabolism. Indeed, inhibition of all JNK isoforms with JNK inhibitor II, also known as SP-600125, dose-dependently suppressed respiration in response to CL-316,243 stimulation (Fig. 4A). Adipocytes express both JNK1 and JNK2 isoforms. Thus, we also tested the effect of JNK inhibitor IX, which actively inhibits JNK2, but not JNK1^25^. Interestingly, no effect of JNK inhibitor IX on lipolysis driven respiration was observed at any of the doses tested (Fig. 4B). Conversely, JNK1 specific inhibition with DB-07268 resulted in a dose dependent suppression of lipolysis-driven oxidative metabolism (Fig. 4C). Consistent with JNK activation by fatty acids, BSA sequestration of fatty acids outside of the adipocyte suppressed the activation of JNK during lipolysis (Fig. 4D). BSA and JNK1 inhibition had an additive effect on JNK phosphorylation levels and lipolysis driven oxidative metabolism (Fig. 4C–F). Consistent with STAT3 phosphorylation as the mechanism by which JNK promotes oxidative metabolism, no effect of JNK inhibition on respiration was observed in the STAT3 knockout adipocytes (Fig. 4G). Together these data suggest that fatty acids activate JNK1 which then phosphorylates STAT3 at Ser^727^ to suppress esterification and promote oxidative metabolism.

**Figure 5:**
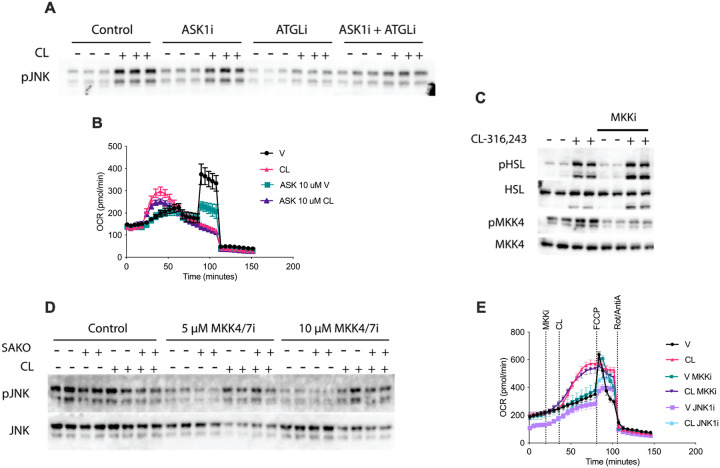
MAP kinase signaling upstream of JNK activation in lipolytic adipocytes. **A** Western blot analysis was performed on treated PPDIVs following stimulation with 100 nM CL-316,243 (CL) or vehicle, 5 μM ASK1i or vehicle, and 50 μM ATGLi or vehicle. **B** OCR in primary adipocytes pretreated with 10 μM ASKi or vehicle, then stimulated with 100 nM CL or vehicle in Port A. **C** Western blot analysis was performed on PPDIV-differentiated adipocytes stimulated with 1 μM CL-316,243 (CL) or vehicle, in addition to 5 μM MKKi or vehicle. **D** Western blot analysis of treated PPDIVs from SAKO and littermate control SAWT mice, following stimulation with1 μM CL-316,243 (CL) or vehicle and 5 μM and 10 μM MKK4/7i or vehicle. **E** Oxygen consumption rate (OCR) in differentiated primary adipocytes, measured from baseline to 30 minutes after stimulation with either 5 μM MKKi or vehicle in Port A. Treatment in Port B: 10 nM CL-316,243 (CL) or vehicle; Treatment in Port C: 1 μM FCCP; Treatment in Port D: 1 μM rotenone/antimycin A (R/A).

Although fatty acid mediated activation of JNK has been observed in multiple cell types and varying conditions, the mechanism is unclear. Inhibition of ATGL blocks lipolysis and prevents the increase in intracellular fatty acids, without suppressing upstream catecholamine signaling. ATGL inhibition effectively blocked JNK1 activation by CL-316,243 consistent with activation by fatty acids not catecholamine signaling. We next investigated the MAP kinase signaling cascade upstream of JNK. The MAP3K Ask1 has been implicated in JNK activation in response to ER stress^26^. Consistent with previous reports that ASK1 does not mediate fatty acid stimulation of JNK^27^, we did not observe an impact of ASK1 inhibition on lipolysis-induced oxidative metabolism in adipocytes (Fig. 5B). MKK4 and MKK7 are the canonical kinases phosphorylating threonine and tyrosine residues in JNK^28^. Phosphorylation of MKK4 in response to lipolytic activation was observed after only 15 minutes and blocked by the MKK4/7 inhibitor BSJ-04–122. While MMK4/7 inhibition suppressed baseline JNK phosphorylation, it did not block JNK activation by CL-316,243 (Fig. 5C and D). Furthermore, the MMK4/7 inhibitor had no impact on lipolysis driven oxidative metabolism, although in the same experiment a defect was observed in the presence of JNK inhibitor II (Fig. 5E). These results suggest that the MAP kinase cascade upstream of JNK activation in lipolytic adipocytes may be atypical.

## Discussion

Here we present data suggesting that JNK1 is the kinase that phosphorylates STAT3 at Ser^727^ in lipolytic adipocytes. JNK activation is mediated by the elevation in intracellular fatty acid levels, however the upstream MAP kinase cascade is unclear. ASK1 and MKK4/7 do not appear to be required. Involvement of the MAP3K, MLK3, would be consistent with the feed forward activation of JNK evidenced by the suppression of JNK phosphorylation by JNK inhibition, however, this remains to be tested.

Another open question is the spatial specificity of STAT3 phosphorylation at the lipid droplet. Lipolytic activation had a dramatic effect on lipid droplet associated proteins. Our data does not preclude the possibility that JNK1 localization to the lipid droplet is increased during lipolysis. However, this would not explain the absence of STAT3 phosphorylation by JNK in other factions where both proteins are found. It is possible that changes in other lipid droplet associated proteins upon lipolysis may promote JNK phosphorylation of STAT3 either due to proximity or accessibility of STAT3 to JNK.

The role of JNK in energy balance and obesity is ambiguous. Elevated fatty acid levels in obese adipose tissue have been associated with JNK activation and phosphorylation of IRS1 leading to insulin resistance^21, 29^. While whole body JNK1 knockout results in resistance to diet induced obesity, adipocyte specific JNK1 knockout does not exhibit this phenotype, despite increased energy expenditure via classic UCP1 mediated thermogenesis^30^. The metabolic impact of JNK is likely related not only to the cell type in which it is activated, but also the metabolic context and duration of activation i.e. chronic versus acute. In the context of adipocyte lipolysis we find that JNK1 promotes energy expenditure. While adding to the complexity of metabolic regulation by JNK, our findings in lipolytic adipocytes are likely unrelated to chronic JNK activation in obese adipose tissue which has been implicated in insulin resistance. The myriad of JNK impacts on metabolism likely contribute to the ambiguity of its overall impact in vivo. These studies were performed in adipocytes isolated from both male and female C57bl6 mice. Relevance in other murine strains and mammalian species must be confirmed experimentally.

## Data Availability

All data are provided within the manuscript

## Abbreviations

WAT: white adipose tissue
JNK1: c-Jun N-terminal kinase 1
STAT3: signal transducer and activator of transcription 3
SAKO: adipocyte-specific knockout
SAWT: floxed littermate control of SAKO
TMRM: tetramethylrhodamine methyl ester
ACSL: acyl CoA synthetases
ASK1: apoptosis signal-regulating kinase 1
DGAT: diglyceride acyltransferase
MKK4/7: mitogen-activated protein kinase kinases 4/7
MAPK: mitogen – activated protein kinase
OCR: oxygen consumption rate
p38: p38 mitogen-activated protein kinase
CL: The beta-3 adrenergic agonist (CL-316,243)
MEK: mitogen-activated protein kinase kinase
ERKs: extracellular signal-regulated kinases
GPAT3: glycerol-3-phosphate acyltransferase 3
DMEM: Dulbecco’s Modified Eagle Medium
